# Supplementary material for: A time-domain phase diagram of metastable states in a charge ordered quantum material
Source: Nat Commun. 2021 Apr 19;12:2323. doi: 10.1038/s41467-021-22646-7 (PMC8055663; doi:10.1038/s41467-021-22646-7)
Supplement: Supplementary file 1 — Supplementary Information [file 41467_2021_22646_MOESM1_ESM.pdf]

Supplementary Information

A time-domain phase diagram of metastable states in a charge ordered quantum material

Jan Ravnik, Michele Diego, Yaroslav Gerasimenko, Yevhenii Vaskivskyi, Igor Vaskivskyi, Tomaz Mertelj, Jaka Vodeb and Dragan Mihailovic[*]

Supplementary note 1. Details of STM experiments

Hidden state. The threshold fluence for switching to the hidden state as seen with the STM appears to change with the temperature (Supplementary Figure 1). At 4 K the average threshold fluence for observing the H state is 1.5 mJ/cm^2, while at 77 K we only observe the H state where the fluence is larger than 5.7 mJ/cm^2 (the observed area of the H state on the sample is smaller for the same laser beam). This is due to the faster relaxation of the H state at higher temperatures, which happens on the outskirts of the beam and prevents us from observing the whole switched area of H state with the relatively slow STM device. The observation could as well imply a higher switching threshold at higher temperatures, but the transient reflectivity measurements do not support this claim. Another important observation is that the observed switching fluence stays independent from the number of the incident pulses, both at 77 K and at 4 K. A very slight decrease in the threshold fluence (increase of the switched area) at 4 K with increasing the number of pulses is most likely the consequence of the spatial drift of the beam during long exposures. We conclude that the laser induced accumulated heating and nonequilibrium photoexcitations are well-separated effects.

Amorphous state. The amorphous state does not appear consistently across all experiments. In some cases, it is observed in patches within a larger area of the H state, while in other cases it is mixed with ISC areas. At 4 K the amorphous state was found both surrounded by H state and mixed with ISC, while when excited at 77 K it was only found mixed with ISC. On heating the A state from 4 K above 77 K, it was found surrounded by the C state when not mixed by ISC. In contrast with the electric switching[1], we have not found a way to

controllably use optics to reproducibly switch the sample to the A state. However, we have found the boundary conditions at which the A state is likely to appear in optical experiments.

Irreversible structural changes. We observe no correlation between the sample temperature and the area of the induced ISC at the sample temperatures of 4 and 77 K (Supplementary Figure 2). However, the area of ISC increases with the number of laser pulses, implying that at large fluences the accumulated heating plays a more critical role as opposed to low fluences. In most cases the threshold fluence to induce ISC is between 7 and 10 mJ/cm^2. This is particularly important for the polytype transformations[2], which were never observed in a single shot experiment, as they are a consequence of heating the sample, rather than an ultrafast electronic process. The same experimental conditions (power, exposure time and temperature) that lead to the polytype transformation in one case can create a large visible hole in the sample in another case. Single shot experiments can also have different ISC outcomes. When switching the sample with a single shot at two well-separated positions on the same crystal, we did not observe any ISC in one case, while in the other case we were able to see ISC in the form of melted sample in the middle. In both experiments the peak fluence exceeded 10 mJ/cm^2 and the sample was held at 4 K. This clearly suggests that the outcomes at high fluences are strongly dependent on local properties of the sample, which are responsible for uneven sample cooling. The heat transfer is determined by the quality of the thermal coupling between the top layer(s) and the bulk sample and/or the sample holder. This varies with the amount of glue, the thickness of the sample and the interlayer structure, which are not very well controlled experimental variables.

Boundaries between the photoinduced states. While the H state is usually found in large homogeneous patches (> 1 μm^2), the A state and ISC more likely appear in smaller patches intermixed with other states. The boundaries between the states are usually gradual. As an example, a transition between an H area and a completely melted sample area generally includes a few intermediate areas. First, transiting from the pure H state, we see the H state

gets mixed with A state areas or covered with lots of small (< 5 nm) material deposits when moving towards the higher fluence region. Further, the ratio of the H area and the melted parts changes and larger melted parts start appearing more frequently (Supplementary Figure 3b and c) until the H parts completely disappear in favor of the melted region. Similar kind of transitions are observed in all cases, many times including spots where more than two states are intermixed (Supplementary Figure 3a).

As a contrast to Figure 1h in the main text, we show Supplementary Figure 3d, where most of the area is transformed to 1H polytype with only small triangles of 1T.

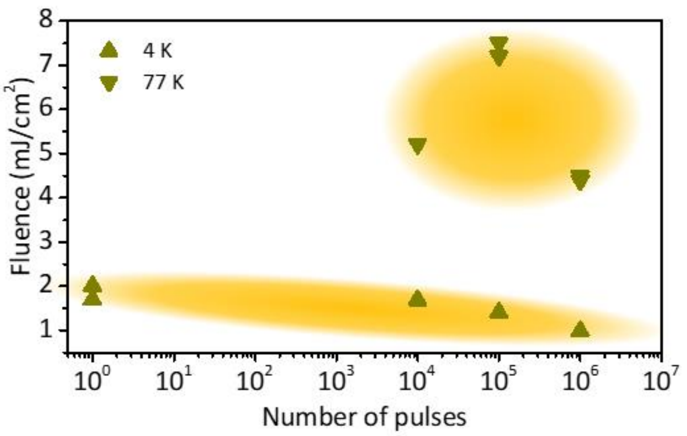

Supplementary Figure 1. Threshold fluence for switching to the hidden state with respect to the number of shots. The upward and downward facing triangles show measurements at 4 K and 77 K respectively. The threshold fluence is lower at 4 K in all cases, which we attribute to the faster relaxation at 77 K. The yellow ellipses serve only as a guide to the eye.

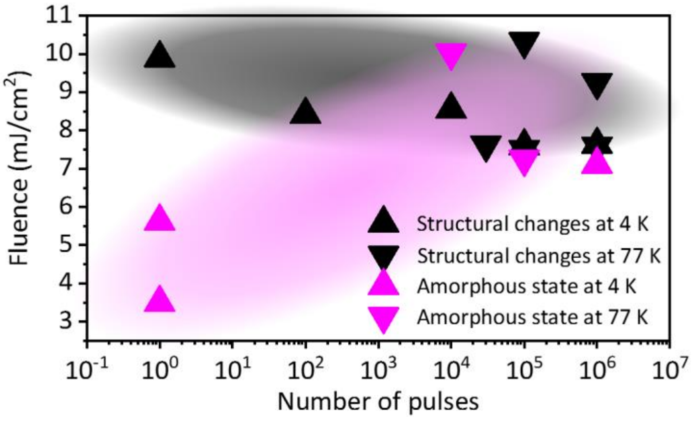

Supplementary Figure 2. The observed threshold fluences for switching to the amorphous state (pink) and to induce structural changes (black) with respect to the number of shots. The upward and downward facing

triangles show measurements at 4 K and 77 K respectively. The black and pink ellipses serve only as a guide to the eye.

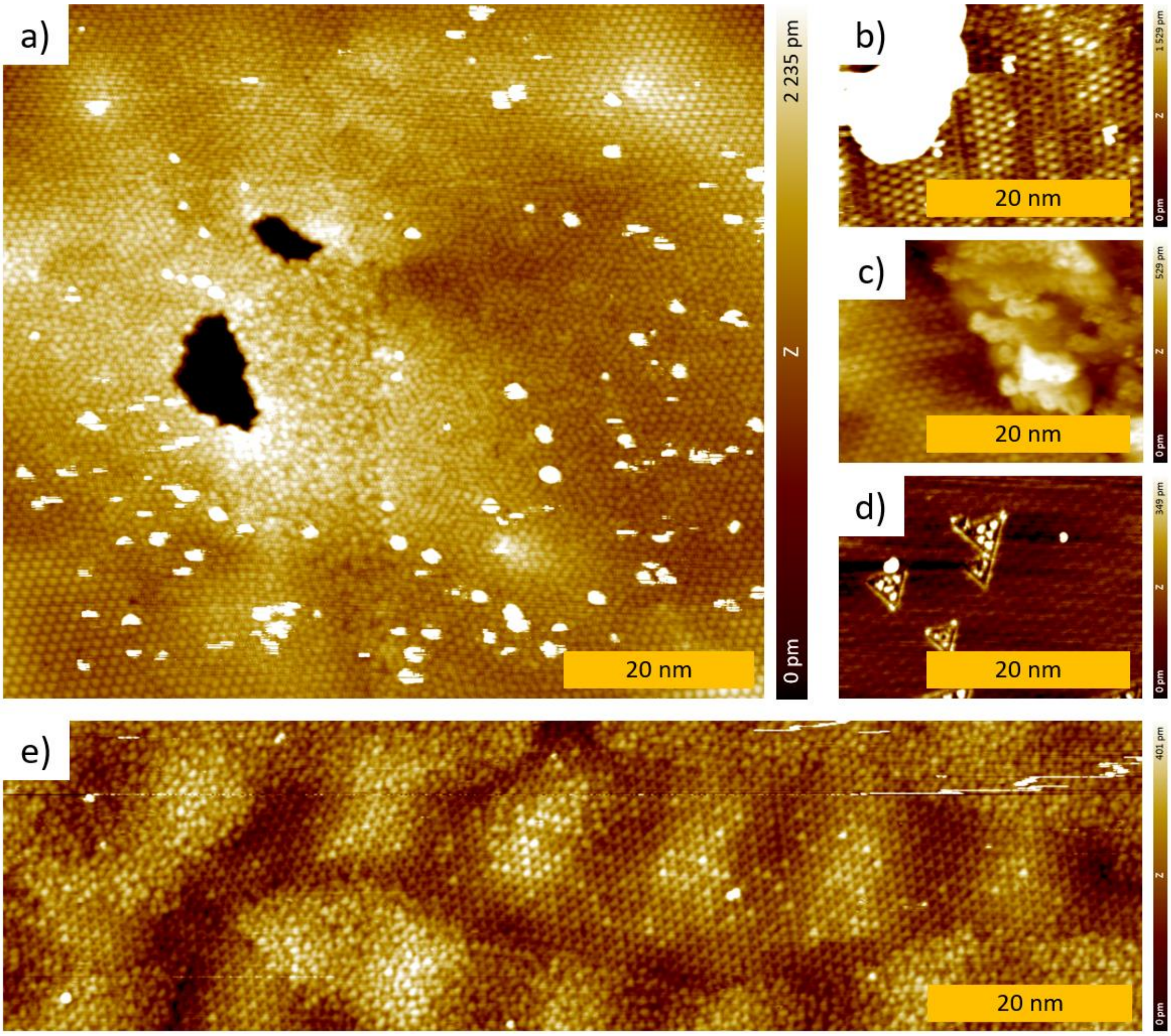

Supplementary Figure 3. STM images of areas with more than one state present. a) Mixture of H and A state with ISC in form of material deposits (white) and holes (black), b) and c) H state with large material deposits, d) 1H polytype area with small triangles of 1T polytype. e) a large A area encapsulating a small H area.

Supplementary note 2. Transient reflectivity measurements

Transient reflectivity at 100 and 160 K. The first set of D-P-p measurements was done at 100 K, with the D-P delays of 400 ps, 30 ps, -3 ps and -30 ps and with the fluences ranging from 0.4 mJ cm^{-2} to 10 mJ/cm^2 (Supplementary Figure 4). In case where the D pulse arrives before the P-p sequence, we performed two separate sets of measurements, for the D-P delay of 30 ps and 400 ps. This way make sure that the observed state is not changing on the

timescale of the measured P-p trace (30 ps). Since the excited states at $t_{D\text{-}P}$ = 30 ps and $t_{D\text{-}P}$ = 400 ps show the same characteristics, we conclude that they are stable on the 10-100 ps timescale and further consider only the measurements at $t_{D\text{-}P}$ = 30 ps.

One can notice the generally decreasing amplitude of the oscillations when increasing the D pulse fluence. Comparing the measurements of the millisecond state to picosecond state, we can see that the oscillation amplitude is at all fluences at least partly restored, suggesting that some sort of relaxation takes place even at the highest fluences. At fluences higher than 10 mJ/cm^2 a visible damage (a hole) was created and we were unable to perform any further measurements on that part of the sample. From the measurements, where the D pulse hits the sample during the P-Pr sequence ($t_{D\text{-}P}$ = -3 ps) we see that above a certain threshold fluence the slow transient reflectivity component (neglecting the oscillatory part) changes to another value. This is associated with the switching to the H state and was described in details previously[3].

To more accurately determine the states, we did fast Fourier transforms (FFT) of the data for the millisecond and picosecond states (at $t_{D\text{-}P}$ = -30 ps (corresponding to the relaxed state after 1 ms) and 30 ps, respectively), which are shown in Supplementary Figure 5. The maximum of the amplitude mode (AM) peak in the millisecond state stays approximately constant for the fluences of up to 3 mJ/cm^2, and corresponds to the C state. Above that fluence the peak becomes broader and weaker, suggesting a change which does not relax back to the initial state within 1 ms. The data for the picosecond state shows a similar change above 3 mJ cm^{-2} as for the millisecond state, but the peaks are even more suppressed than for the millisecond state. The most important thing we see in the picosecond state is that at the fluences above 1 mJ cm^{-2} the AM peak shifts to lower frequencies, indicating the switching to the H state.

An equivalent set of measurements was done also at 160 K. The data shows the same characteristics as at 100 K.

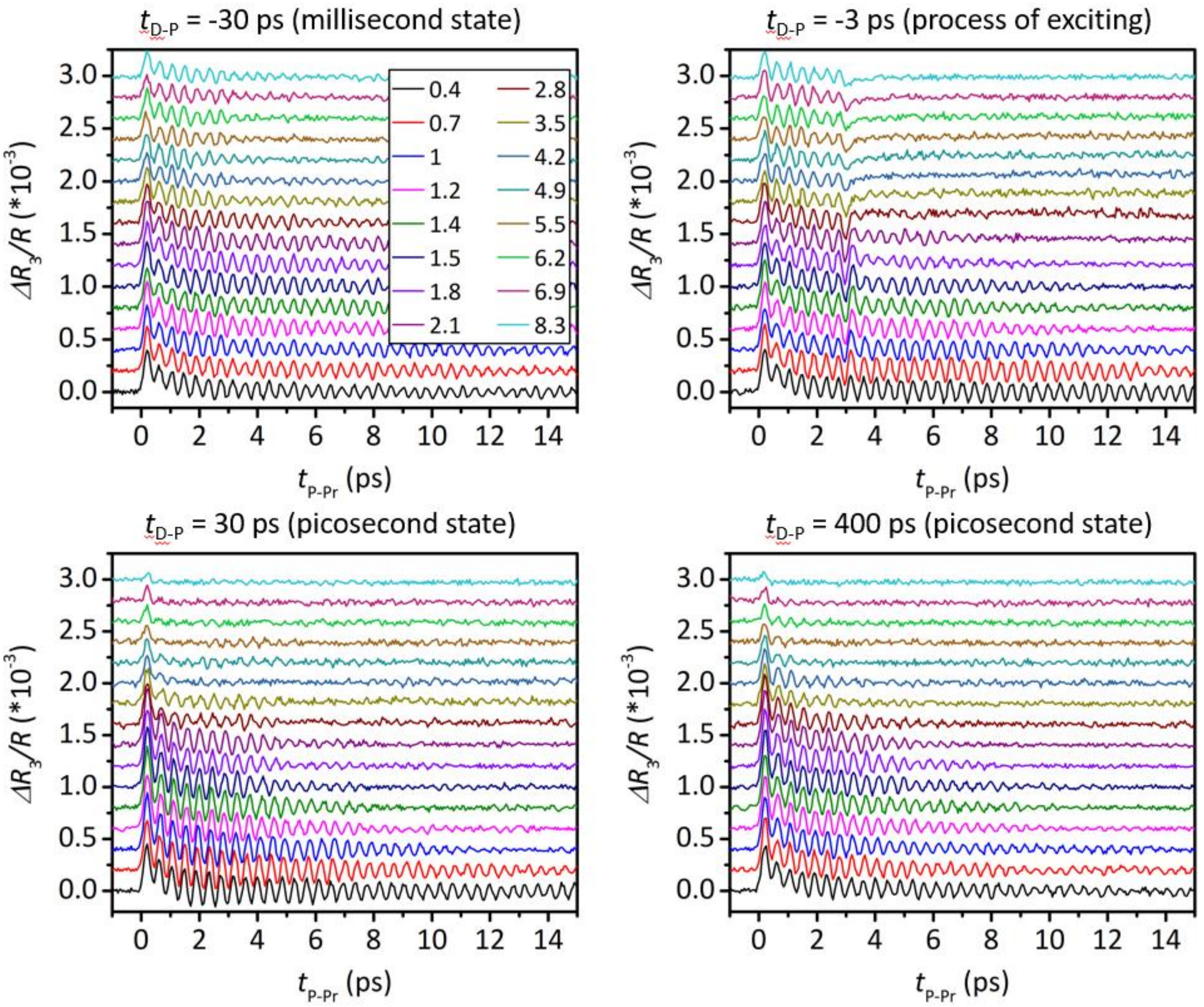

Supplementary Figure 4. Three pulse transient reflectivity data at various D pulse fluences at T = 160 K. The measurements at $t_{D\text{-}P}$ = -30 ps show the sample in a 1 ms state, the measurements at $t_{D\text{-}P}$ = -3 ps show the arrival of D pulse during the P-Pr sequence. The measurements at $t_{D\text{-}P}$ = 30 ps and $t_{D\text{-}P}$ = 400 ps show the excited sample, where the D pulse arrives 30 and 400 ps before the P respectively. We see that the latter two sets show identical behavior. The numbers in the legend represent fluences in mJ/cm^2.

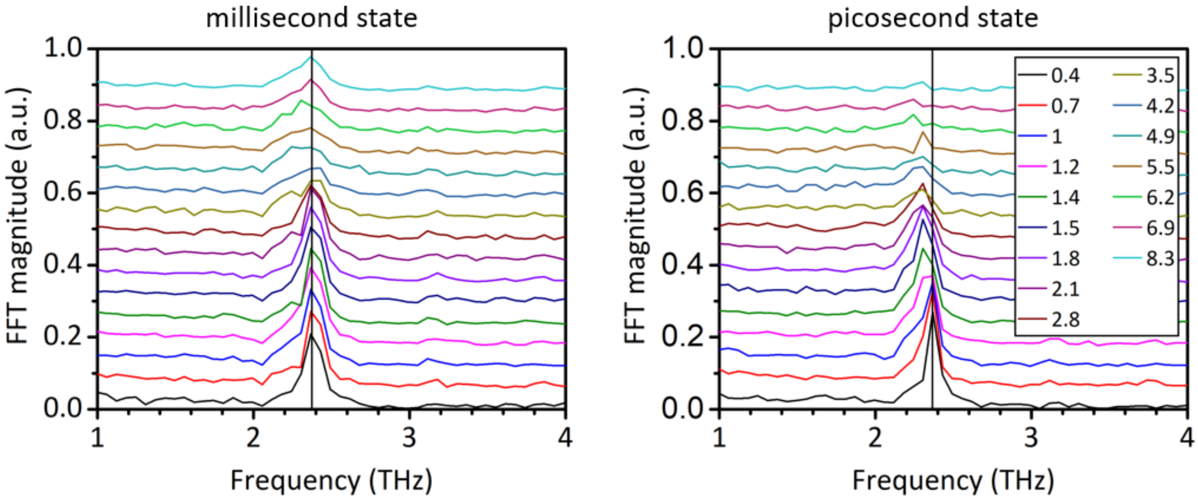

Supplementary Figure 5. AM spectra of the transient reflectivity data at T = 160 K. The data of the millisecond state shows complete relaxation to C state up to the fluence of about 3 mJ/cm^2 and an obvious suppression of the AM peak at higher fluences. The data on the picosecond state shows switching to the H state at the fluence above 1 mJ/cm^2 and a lowering of the peak at fluences above 3 mJ/cm^2. The numbers in the legend represent fluences in mJ/cm^2.

Transient reflectivity at 200 K. At 200 K, 1*T*-TaS_2 can be found in two different thermodynamically stable states, namely the C and NC state, depending on how the sample reached this temperature. Upon cooling it undergoes a phase transition from the NC to C state at 180 K, thus it is in the NC state at 200 K. Upon heating it stays in the C state until 220 K before reaching the triclinic state, thus it is in the C state at 200 K. By performing the experiment at 200 K we check if the switching to the H state can be distinguished from the effects of the accumulated heating of the sample to the triclinic or NC state by the train of D pulses. If the main mechanism is indeed heating, the sample should stay in the NC/triclinic state also after cooling and would not relax back to the C state at this temperature. In this case we would observe the characteristic oscillations of the triclinic or NC state when photoexciting the sample. Another thing that we could expect if heating was the transition mechanism, would be a decrease of the threshold fluence at higher temperatures. The highest measured fluences (up to 1.5 mJ/cm^2) are comparable or higher than the switching fluences observed at lower temperatures, but no change in the transient reflectivity is observed (Supplementary Figure 6). We conclude that switching to the H state is a different process than supercooling a thermodynamically stable state.

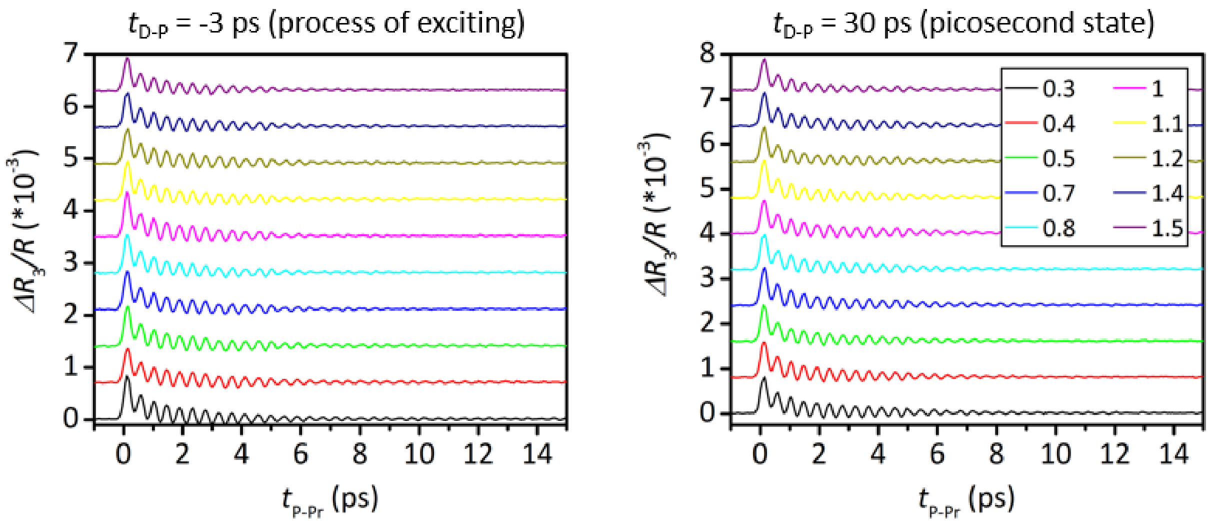

Supplementary Figure 6. Three pulse transient reflectivity for various D pulse fluences at T = 200 K. The graphs show the measurements where the D pulse excites the sample during the P-p measurement (t_{D-P} = -3 ps) and 30 ps before the measurement. The numbers in the legend represent fluences in mJ/cm^2.

Transient reflectivity at 80 K. We performed two-pulse and three-pulse measurements at 80 K, with two different D pulse fluences, in the range where we expected the switching to the H state to occur (2.86 mJ/cm^2) and at a bit higher value (4.41 mJ/cm^2), shown in Supplementary Figure 7. At this temperature, the relaxation time of the H state should be comparable to the time between the laser pulses[4] and thus a two pulse measurement of the C state was also made for the comparison. By exciting the sample with a 2.86 mJ/cm^2 laser pulse, the AM peak moves to a lower frequency with respect to the AM peak in the C state, consistently with observations at other temperatures. When observing the millisecond state, the AM peak seems to be a combination of both C and H AM peaks, suggesting that the sample is only partially relaxed. This is consistent with the STM measurements at 77 K, where the sample partially relaxes. By increasing the fluence to 4.41 mJ/cm^2, we observe that the oscillations in the picosecond state have smaller amplitude consistently with the measurements at higher temperature.

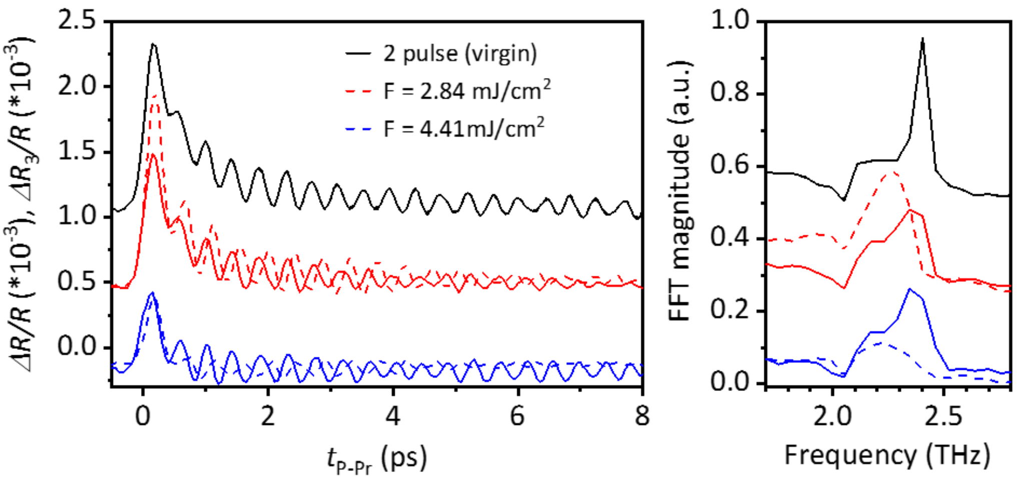

Supplementary Figure 7. Two and three pulse pump probe measurements at 80 K (left) and their respective FFTs (right). The fluence of the D pulse was set to 0 mJ/cm^2 for the two pulse measurement (black) and 2.84 mJ/cm^2 (red) and 4.41 mJ/cm^2 (blue) in three pulse experiments. In the three pulse experiments the dashed and full lines represent the picosecond and millisecond states, respectively. We see that the sample does not relax from H state completely in 1 ms at 80 K. The amplitude of oscillations in the picosecond state significantly drops when the fluence is increased.

Supplementary note 3. Theoretical phase diagram

The parameter range of the filling f, relevant for comparison with the experiment is from $1/14$ to $1/11$, where all the experimentally observed states lie (Figure 3c). The model predicts a polaronic crystalline state (commensurate state) at $f = 1/12, 1/13$. Only $1/13$ is observed experimentally and it corresponds to the thermodynamically stable C state. Upon increasing the value of f from $1/13$, a domain state (experimentally observed H state) emerges, where domains of the $1/13$ lattice are separated by domain walls. Further increasing of f introduces $1/12$ lattice domains into the domain structure, which are not observed experimentally. The $1/13$ domains disappear at some point and only $1/12$ domains are left. When $f = 1/12$ is reached, polarons assume a perfect lattice pattern. Increasing f from $1/12$, a domain state with $1/12$ lattice domains separated by domain walls reemerges, which eventually disappears and we are left with an amorphous state consisting of mixed striped

states[5]. In the experiment, the only values of f observed are $1/13$ in the commensurate state, $\sim 1/12.6$ in the hidden state and $\sim 1/11$ in the amorphous state. To map the model to the experiment, we count the number of polarons per unit area in STM images. We assume the zero doping at the filling level of $1/13$, which corresponds to the equilibrium C state. The deviations from the equilibrium state are linked to photodoping of the system which changes the value of f. The absence of observation of all the possible fillings between the values $1/12.6$ and $1/11$, which includes the $1/12$ lattice as well as the mixture of $1/12$ and $1/13$ lattices is most likely due to various effects, which are not included in the model. The model neglects Fermi surface nesting, electron-phonon coupling, overlapping polaron deformations and itinerant carriers, which are all a part of the complex nature of 1*T*-TaS_2. As an example, Fermi surface nesting is the dominant factor in determining the high temperature incommensurate charge density wave state[6] and could as such significantly influence the outcome of the model. On the other hand, our correlated polaronic approach is valid to a certain degree due to its ability to predict the many different states, which could arise in other similar systems[5].

References

1. Gerasimenko, Y. A. *et al.* Quantum jamming transition to a correlated electron glass in 1T-TaS 2. *Nature materials* **18,** 1078–1083 (2019).

2. Ravnik, J. *et al.* Strain-Induced Metastable Topological Networks in Laser-Fabricated TaS2 Polytype Heterostructures for Nanoscale Devices. *ACS applied nano materials* **2,** 3743–3751 (2019).

3. Ravnik, J., Vaskivskyi, I., Mertelj, T. & Mihailovic, D. Real-time observation of the coherent transition to a metastable emergent state in 1 T- Ta S 2. *Physical Review B* **97,** 075304 (2018).

4. Vaskivskyi, I. *et al.* Controlling the metal-to-insulator relaxation of the metastable hidden quantum state in 1T-TaS2. *Science advances* **1,** e1500168 (2015).

5. Vodeb, J. *et al.* Configurational electronic states in layered transition metal dichalcogenides. *New Journal of Physics* **21,** 083001 (2019).

6. Rossnagel, K. On the origin of charge-density waves in select layered transition-metal dichalcogenides. *Journal of Physics: Condensed Matter* **23,** 213001 (2011).
